# Supplementary material for: Risk-stratification of febrile African children at risk of sepsis using sTREM-1 as basis for a rapid triage test
Source: Nat Commun. 2021 Nov 25;12:6832. doi: 10.1038/s41467-021-27215-6 (PMC8617180; doi:10.1038/s41467-021-27215-6)
Supplement: Supplementary file 1 — Supplementary Information [file 41467_2021_27215_MOESM1_ESM.pdf]

Risk-stratification of febrile African children at risk of sepsis using sTREM-1 as basis for a rapid triage test

Supplementary Information

SUPPLEMENTARY INFORMATION TABLE OF CONTENTS

I. SUPPLEMENTARY TABLES 3

Supplementary Table 1: Baseline characteristics for derivation and validation cohort 3
Supplementary Table 2: Treatments reported at time of presentation 4
Supplementary Table 3: Baseline characteristics between derivation and validation cohorts 5
Supplementary Table 4: Baseline characteristics for children who absconded after 7 days....... 6
Supplementary Table 5: Baseline characteristics for children who were transferred after 7 days 7
Supplementary Table 6: Biomarker geometric means in cohorts combined and derivation and validation cohorts separately. 8
Supplementary Table 7: Performance of 4 biomarkers quantified in all children with available plasma in derivation, primary validation, and secondary validation cohorts 9
Supplementary Table 8: Performance of 4 biomarkers quantified in all children with available plasma in the derivation, primary validation, and secondary validation cohorts in children with and without *P. falciparum* malaria 10
Supplementary Table 9: Likelihood ratios based on sTREM-1 cut-offs from the 10th-99th percentile of distribution in 0.1 increments of the natural logarithm of sTREM-1 in derivation, primary validation, and secondary validation cohorts 11
Supplementary Table 10: False positive and negatives for sTREM-1 cutoffs from the 10th-99th percentile of distribution in 0.1 increments of the natural logarithm of sTREM-1 in derivation, primary validation, and secondary validation cohorts 12
Supplementary Table 11: Studies evaluating plasma sTREM-1 for sepsis diagnosis or outcome 13
Supplementary Table 12: Coefficient of variance for intra-assay performance of 11 plasma biomarkers 15

II. SUPPLEMENTARY FIGURES 16

Supplementary Fig. 1: Biomarker AUROCs for children who died up to 7d or who survived in:... 16
  a. Derivation cohort 16
  b. Validation cohort 16
Supplementary Fig. 2: Distribution of sTREM-1 and probabilities of death for: 17
  a. Derivation Cohort 17
  b. Validation Cohort.......... 18
Supplementary Fig. 3: Calibration plots for Log sTREM-1 values with multiple imputations from: 19
  a. Primary Validation 19
  b. Secondary Validation 19
Supplementary Fig. 4: Calibration plots for sTREM-1 Zones with multiple imputations from:.... 20
  a. Primary Validation 20
  b. Secondary Validation 20
Supplementary Fig. 5: Time-to-event analyses by sTREM-1 zones in the derivation cohort for: . 21
  a. Whole derivation cohort 21
  b. Subgroup of derivation cohort with *P. falciparum* malaria 21
  c. Subgroup of derivation cohort without malaria 21
Supplementary Fig. 6: Time-to-event analyses by sTREM-1 zones in the validation cohort for: . 22
  a. Whole validation cohort 22
  b. Subgroup of validation cohort with *P. falciparum* malaria 22
  c. Subgroup of validation cohort without malaria 22

III. SUPPLEMENTARY REFERENCES 23

I. Supplementary Tables

Supplementary Table 1: Baseline characteristics for derivation and validation cohort

Characteristic at baseline*	Derivation Cohort			Validation Cohort		
	Dead (n=43)	Alive (n=1390)	Odds ratio (95% CI)	Dead (n=52)	Alive (n=1017)	Odds ratio (95% CI)
Age, days	18.1 (13.8)	19.2 (12.9)	0.84 (0.45 to 1.59)	18.3 (12.1)	20.4 (12.9)	0.70 (0.39 to 1.28)
Male (n, %)	25 (58)	760 (55)	1.15 (0.62 to 2.13)	29 (55.8)	562 (55.3)	0.99 (0.56 to 1.74)
Time to MD, hr	1.5 (1.6)	3.0 (2.5)	0.14 (0.05 to 0.37)	1.7 (1.8)	2.9 (2.1)	0.20 (0.09 to 0.45)
Temperature	37.5 (1.5)	37.9 (1.2)	0.50 (0.27 to 0.93)	37.2 (1.1)	37.8 (1.1)	0.33 (0.18 to 0.60)
SpO2%	91.9 (9.5)	97.2 (3.9)	0.37 (0.26 to 0.53)	89.8 (12.5)	96.9 (4.2)	0.33 (0.22 to 0.50)
Heart rate	156 (32)	161 (24)	0.70 (0.38 to 1.27)	164 (33)	161 (24)	1.29 (0.73 to 2.28)
LODS (n, %)						
0	1 (2.3)	846 (60.9)	1.0 (reference)	2 (3.8)	666 (65.5)	1.0 (reference)
1	4 (9.3)	272 (19.6)	12.5 (1.4 to 112.2)	3 (5.8)	165 (16.2)	6.0 (1.0 to 36.5)
2	11 (25.6)	169 (12.2)	55.1 (7.0 to 430.4)	14 (26.9)	118 (11.6)	39.5 (8.8 to 176.3)
3	27 (62.8)	103 (7.4)	221.0 (29.7 to 1647.4)	33 (63.5)	68 (6.7)	161.5 (37.9 to 689.0)
Lactate, mmol/L	7.1 (1.2 to 41.0)	3.3 (0.8 to 14.2)	6.41 (3.53 to 11.64)	7.7 (1.4 to 41.7)	3.3 (0.8 to 13.9)	7.40 (4.16 to 13.17)
P. falciparum Malaria (n, %)	13 (30.2)	746 (53.7)	0.37 (0.19 to 0.72)	24 (46.2)	546 (53.7)	0.74 (0.42 to 1.29)
HIV (n, %)	4 (9.3)	29 (2.1)	4.89 (1.63 to 14.67)	1 (1.9)	13 (1.3)	1.55 (0.20 to 12.26)

Displayed are means (SD), geometric means with 95% reference range (Lactate), or numbers (%).
Derivation cohort included children prospectively consecutively enrolled between February 15, 2012 and October 31, 2012.
Validation cohort included children prospectively consecutively enrolled between November 1, 2012 and August 29, 2013

Supplementary Table 2: Treatments reported at time of presentation

	Intervention	Cohorts combined	Derivation cohort	Validation cohort
**All children**		2502 (100%)	1433 (100%)	1069 (100%)
	Any antimalarial	2003 (81.5%)	1133 (81.3%)	870 (81.8%)
	Any antibiotic	1891 (76.7%)	1091 (77.9%)	800 (75.1%)
	Whole blood transfusion	868 (34.7%)	531 (37.1%)	337 (31.5%)
	IV fluids	298 (12.2%)	213 (15.4%)	85 (8.0%)
	IV glucose	564 (23.0%)	341 (24.6%)	223 (21.0%)
***P. falciparum* Malaria positive**		1329 (100%)	759 (100%)	570 (100%)
	Any antimalarial	1275 (97.0%)	726 (97.3%)	549 (96.7%)
	IV Quinine only	1034 (78.8%)	657 (88.2%)	377 (66.4%)
	Other antimalarial* only	171 (13.0%)	27 (3.6%)	144 (25.4%)
	IV Quinine and other antimalarial	69 (5.3%)	41 (5.5%)	28 (4.9%)
**IMCI pneumonia**		1004 (100%)	611 (100%)	393 (100%)
	Supplemental O_2	110 (11.2%)	55 (9.4%)	55 (14.0%)
	Time to antibiotics (hours)	14.5 (12.7, 17.8)	15.0 (12.3, 18.0)	14.5 (12.8, 17.5)
	Any antibiotic	838 (84.5%)	500 (83.5%)	338 (86.0%)
	Ceftriaxone only	229 (23.1%)	141 (23.5%)	88 (22.4%)
	Other antibiotic# only	342 (34.5%)	220 (36.7%)	122 (31%)
	Ceftriaxone and other antibiotic	267 (26.9%)	139 (23.2%)	128 (32.6%)

Data missingness for intervention variables ranged from 0.02% for whole blood transfusion to 2.4% for IV fluids.
*Antimalarials: Artesunate, Coartem, Artesunate/Coartem combination, Artemether, Sulfadoxine/Pyrimethamine (Fansidar)
#Antibiotics: Ampicillin, Amoxicillin, Penicillin, Cloxacillin, Ampiclox, Cephalexin, Gentamicin, Trimethoprim-Sulfamethoxazole, Chloramphenicol, Metronidazole, Ciprofloxacin

Supplementary Table 3: Baseline characteristics between derivation and validation cohorts

Characteristic at baseline*	Derivation (n=1433)	Validation (n=1069)	Odds ratio (95% CI)
Age, months	19.2 (12.9)	20.3 (12.9)	1.18 (1.01 to 1.39)
Male (n, %)	784 (54.7)	591 (55.3)	1.02 (0.87 to 1.20)
Time to MD, hr	3.0 (2.5)	2.9 (2.1)	0.88 (0.75 to 1.04)
Temperature	37.9 (1.2)	37.8 (1.1)	0.84 (0.72 to 0.99)
SpO2%	97.1 (4.2)	96.6 (5.2)	0.80 (0.68 to 0.95)
Heart rate	160.4 (24.4)	160.8 (24.7)	1.04 (0.88 to 1.22)
LODS (n, %)			
0	847 (59.1)	668 (62.5)	1.00 (reference)
1	276 (19.3)	168 (15.7)	0.77 (0.62 to 0.96)
2	180 (12.6)	131 (12.3)	0.92 (0.72 to 1.18)
3	130 (9.1)	101 (9.4)	0.98 (0.74 to 1.30)
Lactate, mmol/L	3.4 (0.8 to 14.9)	3.4 (0.8 to 15.4)	1.04 (0.88 to 1.22)
P. falciparum Malaria (n, %)	759 (53.0)	570 (53.3)	1.01 (0.87 to 1.19)
HIV (n, %)	35 (2.4)	15 (1.4)	0.56 (0.29 to 1.08)

Displayed are means (SD), geometric means with 95% reference range (Lactate), or numbers (%). Odds ratios are for the comparison of children in derivation and validation cohorts in case of binary or categorical characteristics, and per 2 standard deviations in case of continuous characteristics to allow direct comparison of odds ratios.[1] An odds ratio <1.0 indicates that averages or percentages were lower in the validation cohort.

Derivation cohort included children prospectively consecutively enrolled between February 15, 2012 and October 31, 2012.

Validation cohort included children prospectively consecutively enrolled between November 1, 2012 and August 29, 2013

Supplementary Table 4: Baseline characteristics for children who absconded after 7 days

Characteristic at baseline*	Absconded (n=337)	Discharged (n=2039)	Odds ratio (95% CI)
Age, months	18.1 (12.3)	20.0 (13.0)	0.74 (0.58 to 0.95)
Male (n, %)	183 (54.3)	1122 (55.0)	0.97 (0.77 to 1.23)
Time to MD, hr	3.0 (2.5)	3.0 (2.4)	1.00 (0.79 to 1.26)
Temperature	37.7 (1.2)	37.9 (1.2)	0.74 (0.58 to 0.94)
SpO2%	97.3 (4.0)	97.1 (4.0)	1.10 (0.82 to 1.48)
Heart rate	161.2 (24.3)	160.5 (24.2)	1.06 (0.84 to 1.35)
LODS (n, %)			
0	201 (59.6)	1298 (63.7)	1.00 (reference)
1	53 (15.7)	379 (18.6)	0.91 (0.66 to 1.26)
2	49 (14.5)	232 (11.4)	1.36 (0.96 to 1.91)
3	34 (10.1)	130 (6.4)	1.70 (1.13 to 2.55)
Lactate, mmol/L	3.7 (0.8 to 16.8)	3.2 (0.8 to 13.3)	1.51 (1.19 to 1.91)
P. falciparum Malaria (n, %)	172 (51.0)	1106 (54.2)	0.88 (0.70 to 1.11)
HIV (n, %)	3 (0.9)	38 (1.9)	0.47 (0.15 to 1.45)

Displayed are means (SD), geometric means with 95% reference range (Lactate), or numbers (%). Odds ratios are for the comparison of children who absconded with children who were regularly discharged from hospital or survived up to 7 days in case of binary or categorical characteristics, and per 2 standard deviations in case of continuous characteristics to allow direct comparison of odds ratios.[1] An odds ratio <1.0 indicates that averages or percentages were lower in children who absconded.
Derivation cohort included children prospectively consecutively enrolled between February 15, 2012 and October 31, 2012.
Validation cohort included children prospectively consecutively enrolled between November 1, 2012 and August 29, 2013

Supplementary Table 5: Baseline characteristics for children who were transferred after 7 days

Characteristic at baseline*	Transferred (n=31)	Discharged (n=2039)	Odds ratio (95% CI)
Age, months	19.9 (15.4)	20.0 (13.0)	0.99 (0.49 to 2.01)
Male (n, %)	17 (54.8)	1122 (55.0)	0.99 (0.49 to 2.03)
Time to MD, hr	2.0 (2.0)	3.0 (2.4)	0.33 (0.13 to 0.83)
Temperature	37.5 (0.9)	37.9 (1.2)	0.52 (0.25 to 1.08)
SpO2%	95.9 (5.7)	97.1 (4.0)	0.68 (0.43 to 1.08)
Heart rate	163.7 (21.3)	160.5 (24.2)	1.32 (0.63 to 2.76)
LODS (n, %)			
0	13 (41.9)	1298 (63.7)	1.00 (reference)
1	5 (16.1)	379 (18.6)	1.32 (0.47 to 3.72)
2	6 (19.4)	232 (11.4)	2.58 (0.97 to 6.86)
3	7 (22.6)	130 (6.4)	5.37 (2.10 to 13.71)
Lactate, mmol/L	6.0 (1.0 to 34.5)	3.2 (0.8 to 13.3)	4.59 (2.26 to 9.32)
P. falciparum Malaria (n, %)	14 (45.2)	1106 (54.2)	0.69 (0.34 to 1.42)
HIV (n, %)	0 (0.0)	38 (1.9)	NE

Displayed are means (SD), geometric means with 95% reference range (Lactate), or numbers (%). Odds ratios are for the comparison of children who were transferred with children who were regularly discharged from hospital or survived up to 7 days in case of binary or categorical characteristics, and per 2 standard deviations in case of continuous characteristics to allow direct comparison of odds ratios.[1] An odds ratio <1.0 indicates that averages or percentages were lower in children who were transferred.

Supplementary Table 6: Biomarker geometric means in cohorts combined and derivation and validation cohorts separately.

Biomarker (pg/mL)	Cohorts combined		Derivation cohort		Validation cohort	
	Alive (n=1989)	Dead (n=95)	Alive (n=984)	Dead (n=42)	Alive (n=1005)	Dead (n=53)
	Geometric mean (95% RR)	Geometric mean (95% RR)	Geometric mean (95% RR)	Geometric mean (95% RR)	Geometric mean (95% RR)	Geometric mean (95% RR)
**sTREM-1**	231 (64 to 829)	792 (187 to 3360)	237 (65 to 861)	864 (181 to 4136)	222 (63 to 785)	736 (194 to 2802)
**sFlt1**	394 (53 to 2911)	1499 (138 to 16328)	413 (56 to 3046)	1793 (189 to 16998)	369 (50 to 2719)	1292 (108 to 15423)
**IL-8**	13 (1 to 273)	172 (2 to 18748)	13 (1 to 285)	234 (2 to 25175)	14 (1 to 257)	134 (1 to 14478)
**Ang-2**	8004 (1954 to 32787)	20221 (4522 to 90428)	8093 (1979 to 33098)	23264 (5101 to 106108)	7884 (1920 to 32372)	18007 (4202 to 77165)
**CHI3L1**	44 (4 to 490)	234 (10 to 5454)	46 (4 to 486)	303 (13 to 7102)	42 (4 to 493)	189 (8 to 4233)
**sTNFR1**	3171 (821 to 12254)	7698 (1349 to 43925)	3427 (914 to 12847)	8341 (1417 to 49120)	2853 (729 to 11165)	7204 (1289 to 40266)
**IL-6**	34 (1 to 1260)	471 (2 to 95973)	35 (1 to 1237)	745 (4 to 138289)	32 (1 to 1288)	322 (2 to 66095)
**sICAM-1**	556 (49 to 6275)	898 (67 to 12114)	584 (51 to 6631)	1062 (83 to 13547)	522 (47 to 5807)	782 (56 to 10950)
**sVCAM-1**	3910 (805 to 19004)	6042 (973 to 37525)	4107 (885 to 19057)	7059 (925 to 53894)	3664 (716 to 18744)	5313 (1061 to 26605)
**IP-10**	13 (1 to 273)	172 (2 to 18748)	13 (1 to 285)	234 (2 to 25175)	14 (1 to 257)	134 (1 to 14478)
**Ang-1**	2606 (241 to 28204)	1300 (130 to 12963)	2793 (265 to 29473)	1433 (111 to 18566)	2378 (215 to 26312)	1200 (151 to 9508)

Data displayed for children with complete follow up and available plasma (n=2084) without imputed data.
RR: Reference range
Derivation cohort included children prospectively consecutively enrolled between February 15, 2012 and October 31, 2012.
Validation cohort included children prospectively consecutively enrolled between November 1, 2012 and August 29, 2013

Supplementary Table 7: Performance of 4 biomarkers quantified in all children with available plasma in derivation, primary validation, and secondary validation cohorts

Biomarker	Derivation (n=1406)	Primary validation (n=1406)	Secondary validation (n=1054)
	AUROC (95% CI)	AUROC (95% CI)	AUROC (95% CI)
**sTREM-1**	0.875 (0.826 to 0.924)	0.876 (0.825 to 0.928)	0.885 (0.841 to 0.929)
**sFlt1**	0.848 (0.780 to 0.917)	0.846 (0.783 to 0.908)	0.786 (0.718 to 0.853)
**Ang-2**	0.832 (0.775 to 0.889)	0.836 (0.776 to 0.896)	0.778 (0.711 to 0.844)
**sTNFR1**	0.777 (0.690 to 0.863)	0.771 (0.696 to 0.846)	0.784 (0.707 to 0.861)

AUROC: Area under the receiver operating characteristic curve; CI: confidence interval. Analyses based on multiple imputation to account for missing vital status in children who were transferred or absconded before 7 days, with all baseline characteristics and in-hospital death up to 7 days as variables in the imputation model to create 20 imputed datasets.
Primary validation was performed in the derivation cohort based on 500 bootstrap samples with replacement).
Secondary validation was performed in the temporally defined validation cohort.

Supplementary Table 8: Performance of 4 biomarkers quantified in all children with available plasma in the derivation, primary validation, and secondary validation cohorts in children with and without *P. falciparum* malaria

Biomarker	Derivation	Primary validation	Secondary validation
	AUROC (95% CI)	AUROC (95% CI)	AUROC (95% CI)
***P. falciparum* malaria positive**	n=746	n=746	n=565
**sTREM-1**	0.924 (0.850 to 0.998)	0.924 (0.849 to 0.998)	0.931 (0.898 to 0.964)
**sFlt1**	0.948 (0.884 to 1.011)	0.948 (0.886 to 1.010)	0.875 (0.821 to 0.928)
**Ang-2**	0.849 (0.754 to 0.944)	0.851 (0.757 to 0.944)	0.857 (0.807 to 0.907)
**sTNFR1**	0.846 (0.736 to 0.956)	0.844 (0.733 to 0.954)	0.851 (0.791 to 0.910)
***P. falciparum* malaria negative**	n=660	n=660	n=489
**sTREM-1**	0.876 (0.812 to 0.939)	0.876 (0.816 to 0.937)	0.871 (0.821 to 0.922)
**sFlt1**	0.860 (0.789 to 0.932)	0.861 (0.794 to 0.928)	0.826 (0.767 to 0.884)
**Ang-2**	0.831 (0.757 to 0.904)	0.833 (0.762 to 0.904)	0.781 (0.719 to 0.843)
**sTNFR1**	0.806 (0.716 to 0.895)	0.805 (0.719 to 0.892)	0.789 (0.720 to 0.857)

AUROC: Area under the receiver operating characteristic curve; CI: confidence interval. Analyses based on multiple imputation to account for missing vital status in children who were transferred or absconded before 7 days, with all baseline characteristics and in-hospital death up to 7 days as variables in the imputation model to create 20 imputed datasets.
Primary validation was performed in the derivation cohort based on 500 bootstrap samples with replacement).
Secondary validation was performed in the temporally defined validation cohort.

Supplementary Table 9: Likelihood ratios based on sTREM-1 cut-offs from the 10th-99th percentile of distribution in 0.1 increments of the natural logarithm of sTREM-1 in derivation, primary validation, and secondary validation cohorts

Interval (pg/mL)	Positive Likelihood Ratio			Negative Likelihood Ratio		
	Derivation (n=1406)	Primary validation (n=1406)	Secondary validation (n=1054)	Derivation (n=1406)	Primary validation (n=1406)	Secondary validation (n=1054)
108	1.2 (1.1 to 1.2)	1.1 (1.1 to 1.1)	1.2 (1.1 to 1.2)	0.13 (0.02 to 0.92)	0.04 (0.00 to 0.20)	0.15 (0.02 to 1.19)
119	1.2 (1.2 to 1.2)	1.2 (1.1 to 1.2)	1.2 (1.2 to 1.3)	0.12 (0.02 to 0.92)	0.03 (0.00 to 0.14)	0.14 (0.02 to 0.90)
132	1.3 (1.2 to 1.3)	1.2 (1.1 to 1.3)	1.3 (1.2 to 1.4)	0.10 (0.01 to 0.72)	0.13 (0.00 to 0.39)	0.11 (0.02 to 0.63)
146	1.4 (1.3 to 1.4)	1.3 (1.2 to 1.3)	1.4 (1.4 to 1.5)	0.09 (0.01 to 0.57)	0.11 (0.00 to 0.32)	0.09 (0.02 to 0.49)
161	1.5 (1.4 to 1.6)	1.4 (1.3 to 1.4)	1.6 (1.5 to 1.7)	0.08 (0.01 to 0.55)	0.10 (0.00 to 0.27)	0.07 (0.01 to 0.41)
178	1.6 (1.5 to 1.7)	1.5 (1.4 to 1.6)	1.7 (1.6 to 1.9)	0.08 (0.01 to 0.44)	0.09 (0.00 to 0.25)	0.12 (0.02 to 0.59)
197	1.8 (1.7 to 2.0)	1.6 (1.5 to 1.7)	2.0 (1.8 to 2.1)	0.07 (0.01 to 0.40)	0.08 (0.00 to 0.22)	0.12 (0.03 to 0.40)
217	2.1 (1.9 to 2.2)	1.8 (1.7 to 2.0)	2.2 (2.0 to 2.5)	0.06 (0.01 to 0.34)	0.08 (0.00 to 0.20)	0.13 (0.04 to 0.38)
240	2.3 (2.1 to 2.5)	2.1 (1.9 to 2.3)	2.6 (2.3 to 2.9)	0.12 (0.02 to 0.56)	0.07 (0.00 to 0.18)	0.11 (0.04 to 0.32)
266	2.7 (2.4 to 3.0)	2.3 (2.1 to 2.6)	3.1 (2.8 to 3.5)	0.14 (0.05 to 0.41)	0.12 (0.00 to 0.26)	0.13 (0.05 to 0.35)
293	3.0 (2.7 to 3.4)	2.7 (2.3 to 3.0)	3.7 (3.2 to 4.2)	0.18 (0.07 to 0.43)	0.15 (0.01 to 0.28)	0.17 (0.07 to 0.39)
324	3.5 (3.0 to 4.0)	3.0 (2.6 to 3.4)	4.5 (3.8 to 5.2)	0.20 (0.09 to 0.42)	0.18 (0.05 to 0.32)	0.18 (0.09 to 0.36)
358	3.8 (3.2 to 4.4)	3.5 (3.0 to 4.1)	5.1 (4.3 to 6.0)	0.31 (0.16 to 0.61)	0.20 (0.06 to 0.34)	0.28 (0.14 to 0.52)
396	4.2 (3.4 to 5.2)	3.8 (3.0 to 4.5)	5.9 (4.8 to 7.3)	0.38 (0.23 to 0.63)	0.32 (0.16 to 0.48)	0.27 (0.17 to 0.44)
438	5.4 (4.3 to 6.8)	4.2 (3.3 to 5.2)	7.0 (5.6 to 8.7)	0.37 (0.24 to 0.56)	0.39 (0.23 to 0.55)	0.32 (0.20 to 0.51)
484	6.7 (5.2 to 8.5)	5.4 (4.1 to 6.7)	7.6 (5.9 to 9.7)	0.40 (0.27 to 0.60)	0.37 (0.22 to 0.53)	0.39 (0.26 to 0.59)
535	8.0 (6.1 to 10.5)	6.7 (4.9 to 8.5)	9.9 (7.5 to 13.2)	0.41 (0.28 to 0.60)	0.41 (0.26 to 0.56)	0.40 (0.28 to 0.57)
591	10.2 (7.6 to 13.7)	8.0 (5.7 to 10.3)	11.4 (8.3 to 15.7)	0.41 (0.28 to 0.59)	0.42 (0.27 to 0.57)	0.43 (0.31 to 0.61)
653	11.2 (8.2 to 15.3)	10.2 (7.0 to 13.3)	10.9 (7.7 to 15.5)	0.48 (0.34 to 0.68)	0.41 (0.27 to 0.56)	0.55 (0.40 to 0.75)
722	12.2 (8.6 to 17.4)	11.4 (7.4 to 15.4)	12.6 (8.4 to 18.9)	0.54 (0.39 to 0.73)	0.48 (0.33 to 0.63)	0.56 (0.44 to 0.72)
798	12.7 (8.5 to 18.8)	12.3 (7.6 to 17.1)	12.8 (8.3 to 19.8)	0.59 (0.45 to 0.78)	0.54 (0.39 to 0.68)	0.58 (0.45 to 0.74)
882	16.4 (10.6 to 25.3)	13.0 (7.4 to 18.6)	12.5 (7.9 to 19.6)	0.61 (0.48 to 0.77)	0.59 (0.45 to 0.73)	0.68 (0.54 to 0.86)
974	20.5 (12.7 to 33.1)	17.1 (8.9 to 25.3)	13.0 (7.6 to 22.4)	0.62 (0.49 to 0.78)	0.60 (0.47 to 0.74)	0.78 (0.65 to 0.94)
1077	23.1 (13.8 to 38.8)	21.8 (9.5 to 34.1)	15.4 (7.8 to 30.4)	0.67 (0.53 to 0.83)	0.62 (0.48 to 0.76)	0.82 (0.71 to 0.94)
1190	26.0 (14.5 to 46.3)	24.9 (9.1 to 40.7)	16.6 (7.5 to 36.4)	0.72 (0.59 to 0.88)	0.66 (0.53 to 0.79)	0.85 (0.74 to 0.96)
1315	17.3 (8.9 to 33.7)	28.4 (7.6 to 49.2)	18.9 (7.6 to 46.9)	0.82 (0.69 to 0.97)	0.72 (0.59 to 0.84)	0.88 (0.78 to 0.99)
1454	22.7 (10.7 to 48.3)	19.0 (3.6 to 34.4)	22.3 (7.7 to 64.7)	0.87 (0.76 to 0.99)	0.81 (0.70 to 0.92)	0.88 (0.79 to 0.97)

Supplementary Table 10: False positive and negatives for sTREM-1 cutoffs from the 10th-99th percentile of distribution in 0.1 increments of the natural logarithm of sTREM-1 in derivation, primary validation, and secondary validation cohorts

Interval (pg/mL)	False Positive			False negative		
	Derivation (n=1406)	Primary validation (n=1406)	Secondary validation (n=1054)	Derivation (n=1406)	Primary validation (n=1406)	Secondary validation (n=1054)
108	86.3%	86.4%	84.0%	0.0%	0.0%	0.1%
119	82.5%	82.5%	80.0%	0.0%	0.0%	0.1%
132	78.9%	78.9%	74.7%	0.1%	0.1%	0.2%
146	73.5%	73.5%	70.4%	0.1%	0.1%	0.2%
161	68.8%	68.8%	63.2%	0.1%	0.1%	0.2%
178	62.5%	62.5%	57.7%	0.1%	0.1%	0.2%
197	57.5%	57.5%	51.4%	0.1%	0.1%	0.3%
217	50.8%	50.7%	45.1%	0.1%	0.1%	0.4%
240	44.6%	44.6%	39.3%	0.1%	0.1%	0.4%
266	38.5%	38.5%	33.3%	0.3%	0.3%	0.4%
293	32.8%	32.8%	27.5%	0.4%	0.4%	0.5%
324	28.1%	28.1%	22.3%	0.5%	0.5%	0.7%
358	23.4%	23.3%	18.1%	0.6%	0.6%	0.8%
396	19.1%	19.0%	14.3%	0.9%	0.9%	1.3%
438	15.5%	15.4%	12.3%	1.2%	1.2%	1.3%
484	12.0%	12.0%	9.7%	1.2%	1.2%	1.6%
535	9.2%	9.1%	8.0%	1.4%	1.4%	2.0%
591	7.5%	7.5%	5.9%	1.4%	1.4%	2.1%
653	5.8%	5.8%	4.9%	1.4%	1.4%	2.3%
722	4.7%	4.7%	4.1%	1.7%	1.7%	2.9%
798	3.8%	3.8%	3.4%	1.9%	1.9%	3.0%
882	3.3%	3.2%	3.3%	2.1%	2.1%	3.1%
974	2.4%	2.4%	2.5%	2.2%	2.2%	3.7%
1077	1.8%	1.8%	1.7%	2.3%	2.2%	4.3%
1190	1.4%	1.4%	1.2%	2.4%	2.4%	4.4%
1315	1.1%	1.1%	0.9%	2.6%	2.6%	4.6%
1454	1.1%	1.1%	0.6%	3.0%	3.0%	4.8%

Supplementary Table 11: Studies evaluating plasma sTREM-1 for sepsis diagnosis or outcome

Study	Study type	Country	Entry criteria	Number of children	Age group	Mortality	sTREM-1 cut-off (pg/mL)	Sepsis AUROC (95% CI)	Mortality AUROC (95% CI)	sTREM-1 assay
Pontrelli, 2016[2]	SR	N/A	Variable	961	Neonates and children	N/A	N/A	-	-	N/A
Bellos, 2018[3]	MA	N/A	Variable	667	Neonates	N/A	N/A	0.95 (0.81–0.99)	-	N/A
Saldir, 2015[4]*	PCS	Turkey	Deterioration due to possible sepsis in NICU	60	Neonates >34wk GA	0%	450	0.97 (0.93-1.0)	-	Quantikine ELISA, R&D
Stein, 2015[5]*	PCS	Israel	ER presentation with suspected SBI	112	Neonates (<3 months)	Not reported	600	0.61 (0.47-0.74)	-	Quantikine ELISA, R&D
Mazzucchelli, 2013[6]*	PCS	Italy	NICU, d5-15 of life, 16 children with and 16 without LONS d16-25	32	Preterm infants <32wk GA	Not reported	N/A	0.80 (0.63-0.92)	-	Flow cytometry, ELISA, R&D
Schlapbach, 2013[7]*	POS	Switzerland	NICU admission with suspected EOS	137	Neonates >34wk GA	0%	1250	0.54 (0.41-0.67)	-	Duoset ELISA, R&D
Sarafidis, 2010[8]*	PCS	Greece	Suspected LONS in NICU	52	Neonates	15.4%	143.35	0.73 (0.59-0.88)	-	Quantikine ELISA, R&D
Chen, 2008[9]	PCS	Taiwan	Hospital admission with suspected SBI	44	Neonates (<3 months)	Not reported	24400	0.88	-	Not reported
Carrol, 2009[10]	PCS	Malawi	ER presentation with pneumonia or meningitis	377	2 months-16 years	22.0%	25000	0.52 (0.43-0.61)	0.54 (0.44-0.64)	Quantikine ELISA, R&D
Kevan, 2011[11]	PCS	USA	Gastrointestinal disease requiring TPN/CVC	24	3 months-4 years	16.7%	N/A	-	-	Quantikine ELISA, R&D
Miedema, 2011[12]	PCS	Holland	Oncology patients on chemotherapy with fever and ANC <500/mm^3	29	1-15 years	Not reported	N/A	-	-	ELISA, unknown manufacturer
Arzanian, 2011[13]	PCS	Iran	Hematology ward admission with malignancy, fever and ANC <500/mm^3	65	15 months-15 years	4.6%	525	0.97	-	Duoset ELISA, R&D
Arizaga-Ballesteros, 2015[14]	PCS	Mexico	Suspected LONS in NICU	71	Neonates	7.0%	300	-	0.88 (0.73-1.00)	ELISA, ab100659, Abcam

Adly, 2014[15]*	PCS	Egypt	112 confirmed sepsis in NICU, 40 healthy newborns	152	Neonates	24.1%	310 (diagnosis) 1100 (mortality)	1.00 (0.70-1.02)	0.98 (0.85-1.13)	Quantikine ELISA, R&D
Erdman, 2011[16]	CCS	Uganda	ER presentation with severe malaria	103	6 months-12 years	22.3%	289·9	-	0·76 (0.66-0.84)	Duoset ELISA, R&D

*Considered for inclusion in meta-analysis by Bellos, *et al.*[3] Sequence of studies corresponds to the sequence presented in the Research in Context section. SR: Systematic Review; MA: Meta-analysis; PCS: Prospective cohort study; CCS: Case-control study; NICU: Neonatal Intensive Care Unit; LONS: Late onset neonatal sepsis (>72h after birth); ER: Emergency room; SBI: Serios bacterial infection; EOS: Early onset sepsis (<72h of birth); GA: Gestational Age; N/A: not applicable; TPN: Total Parenteral Nutrition; CVC: Central Venous Catheter; ANC: Absolute Neutrophil Count

Supplementary Table 12: Coefficient of variance for intra-assay performance of 11 plasma biomarkers

Biomarker	N	Coefficient of Variation (95% CI)
**sTREM-1**	156	2.77 (2.42 to 3.12)
**sFlt1**	156	2.34 (2.00 to 2.68)
**IL-8**	153	2.61 (2.08 to 3.14)
**Ang-2**	176	2.24 (1.98 to 2.50)
**CHI3L1**	152	3.03 (2.51 to 3.55)
**sTNFR1**	177	2.75 (2.24 to 3.26)
**IL-6**	156	3.02 (2.38 to 3.66)
**sICAM-1**	177	3.40 (2.98 to 3.82)
**sVCAM-1**	177	2.24 (1.98 to 2.50)
**IP-10**	176	2.84 (2.41 to 3.27)
**Ang-1**	176	1.77 (1.53 to 2.01)

Displayed are mean coefficients of variation with 95% confidence intervals. Biomarkers were measured using the Luminex multiplex platform in samples from 152 to177 patients; biomarker values outside the dynamic range of the assays were excluded for each biomarker.

II. Supplementary Figures

Supplementary Fig. 1: Biomarker AUROCs for children who died up to 7d or who survived in:

a. Derivation cohort

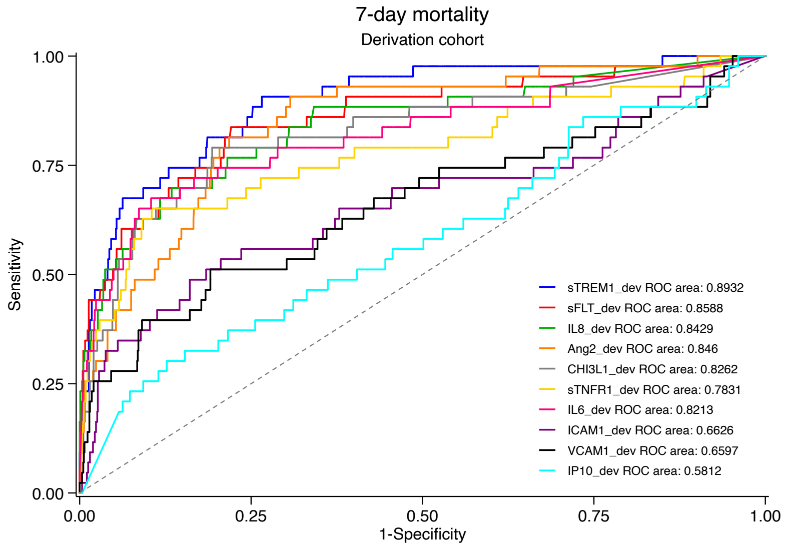

b. Validation cohort

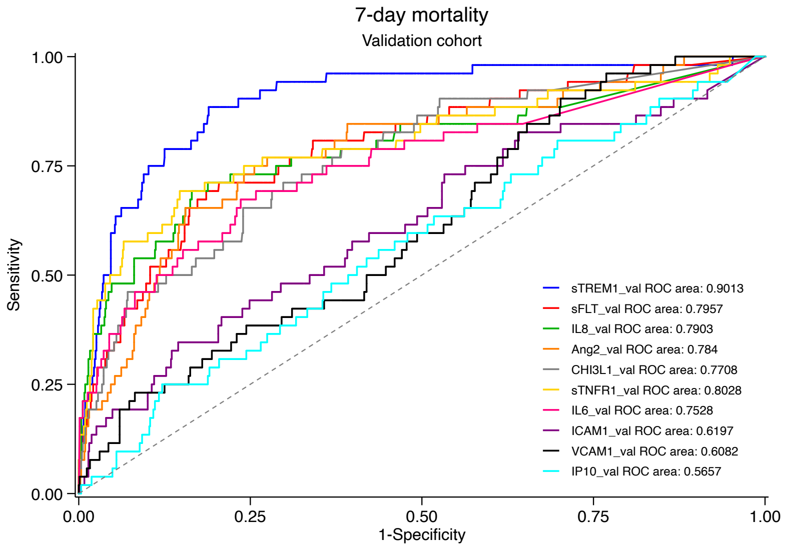

*AUROC curves represent all biomarkers quantified in the (A) derivation (n=1176), and (B) validation (n=908) sets of the complete cohort in children included in biomarker comparative performance (data correspond to AUROC and 95% CI presented in main manuscript Table 2). Derivation cohort included children enrolled between February 15, 2012 and October 31, 2012. Validation cohort included children enrolled between November 1, 2012 and August 29, 2013.

Supplementary Fig. 2: Distribution of sTREM-1 and probabilities of death for:

a. **Derivation Cohort**

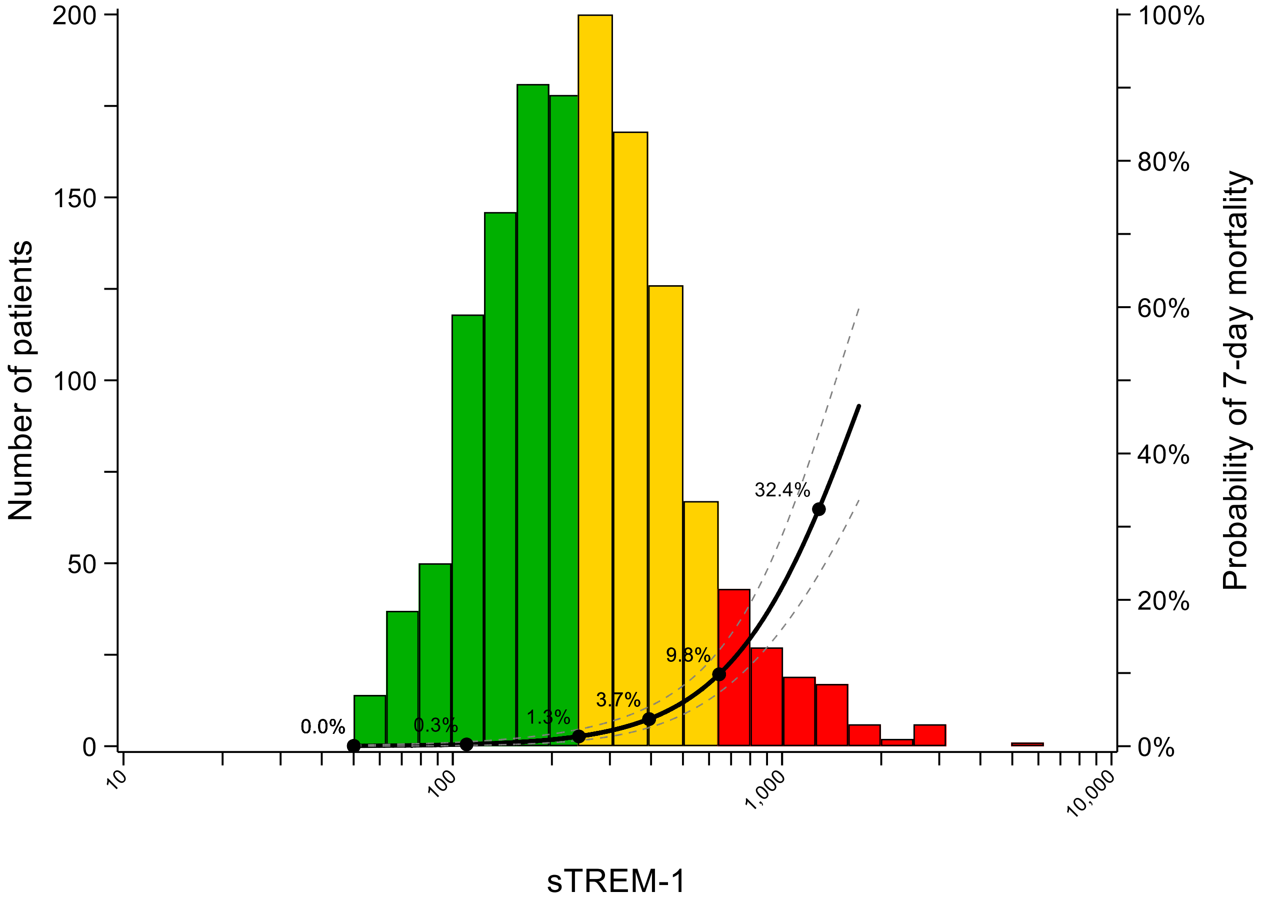

b. Validation Cohort

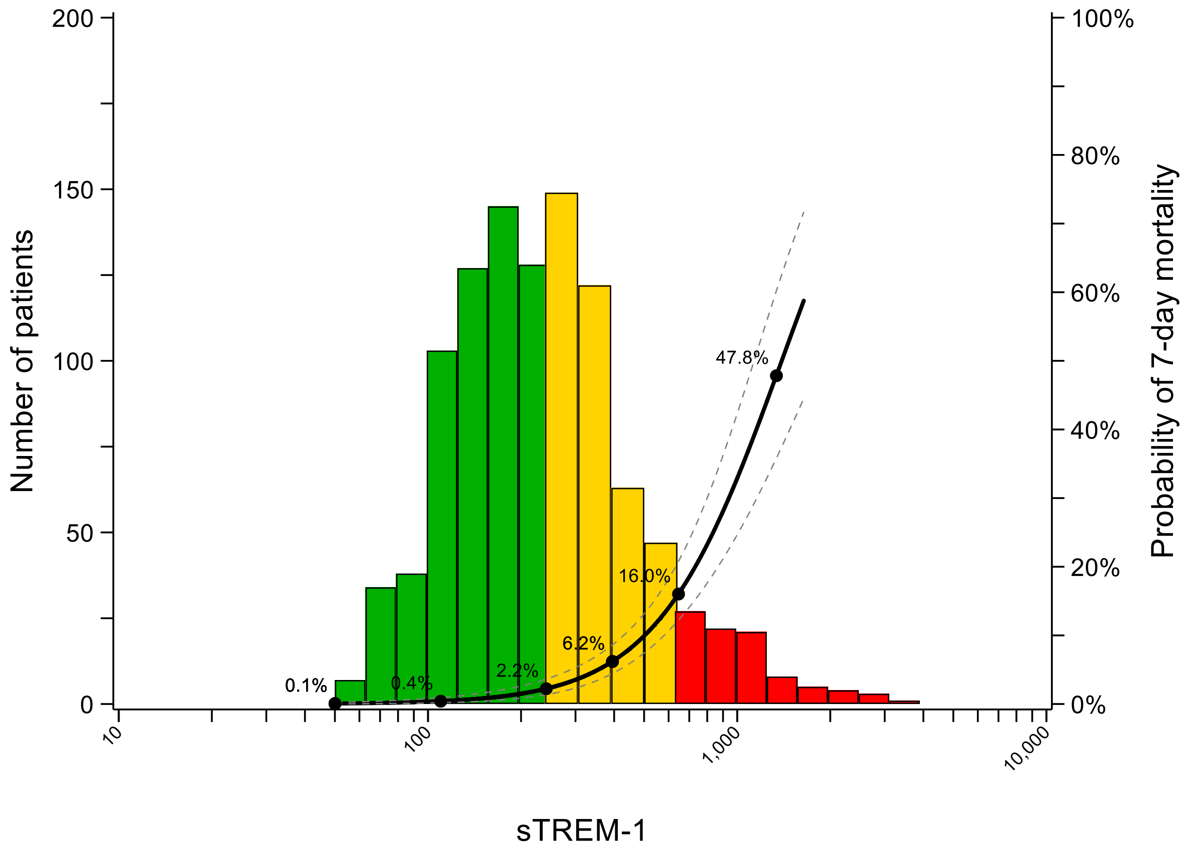

Distribution of sTREM-1 at presentation with predicted probability of 7-day mortality in the (a) derivation and (b) validation cohort. Histogram refers sTREM-1 distribution. Negative and positive Likelihood Ratios (LRs) in the derivation and validation cohorts combined were used to risk-stratify febrile children: “green” zone: low risk (LR- of 0.10, sTREM-1 <239 pg/mL), “yellow” zone: refer and monitor (sTREM-1 ≥239 pg/mL and <629 pg/mL), “red” zone: urgent admission/support (LR+ of 10, sTREM-1 ≥629 pg/mL). Derivation cohort included children enrolled between February 15, 2012 and October 31, 2012.
Validation cohort included children enrolled between November 1, 2012 and August 29, 2013.

Supplementary Fig. 3: Calibration plots for Log sTREM-1 values with multiple imputations from:

a. **Primary Validation**

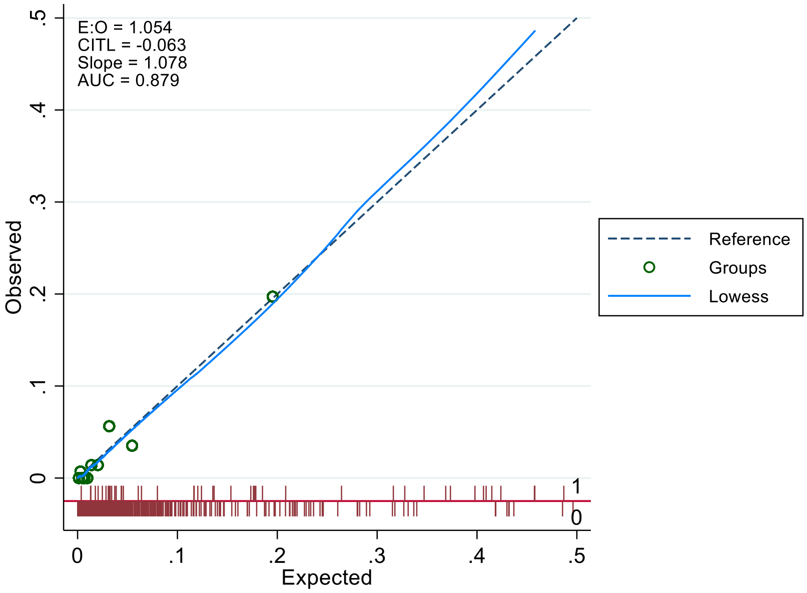

b. **Secondary Validation**

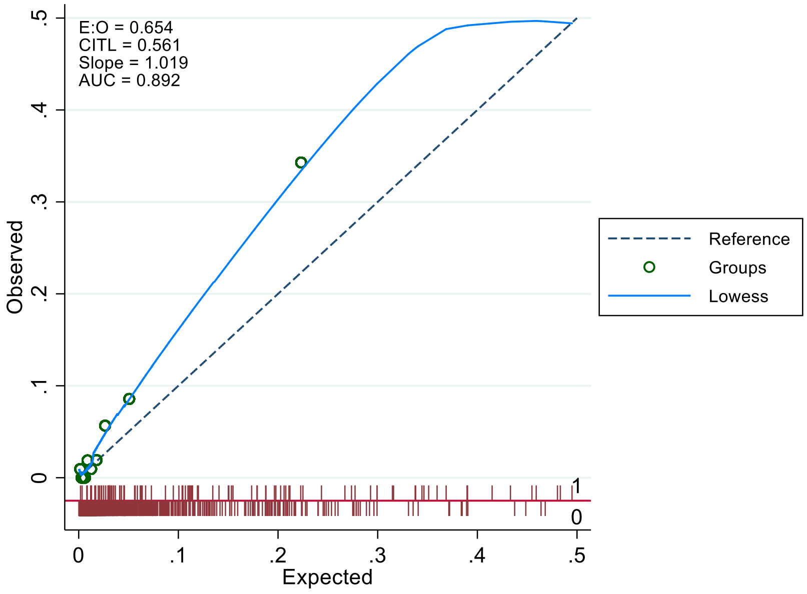

Calibration was defined as the agreement between observed and predicted mortality risk and assessed in calibration plots and calibration-in-the-large.[17] Primary validation was performed in the derivation cohort based on 500 bootstrap samples with replacement). Secondary validation was performed in the temporally defined validation cohort.

Abbreviations: E:O, ratio of expected to observed events for model calibration; CITL, calibration-in-the-large; AUC, Area under the curve

Supplementary Fig. 4: Calibration plots for sTREM-1 Zones with multiple imputations from:

a. Primary Validation

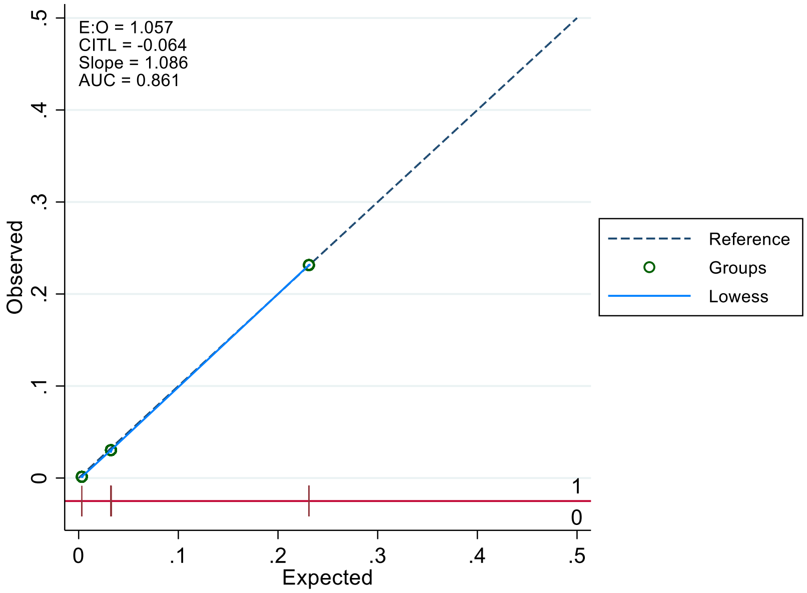

b. Secondary Validation

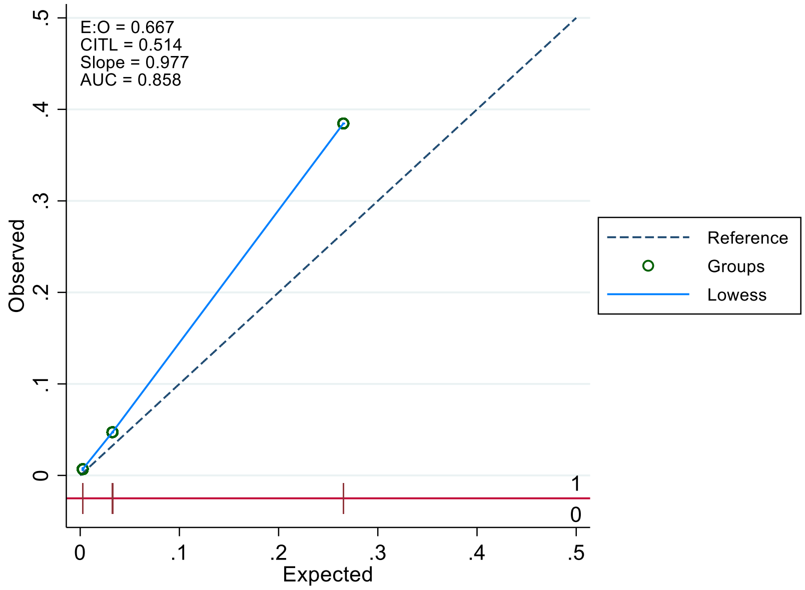

Calibration was defined as the agreement between observed and predicted mortality risk and assessed in calibration plots and calibration-in-the-large.[17] Primary validation was performed in the derivation cohort based on 500 bootstrap samples with replacement). Secondary validation was performed in the temporally defined validation cohort.

Abbreviations: E:O, ratio of expected to observed events for model calibration; CITL, calibration-in-the-large

Supplementary Fig. 5: Time-to-event analyses by sTREM-1 zones in the derivation cohort for:

a. Whole derivation cohort

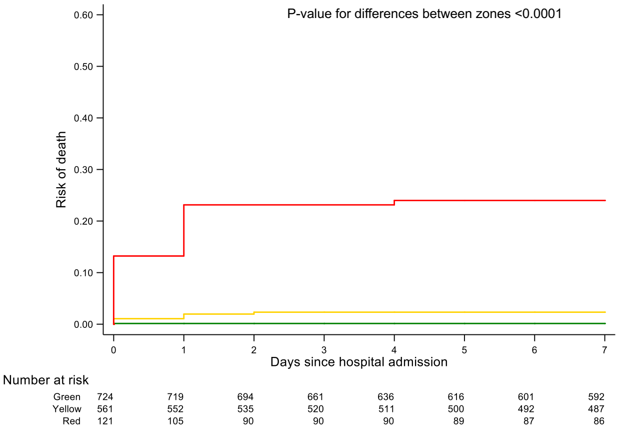

b. Subgroup of derivation cohort with *P. falciparum* malaria

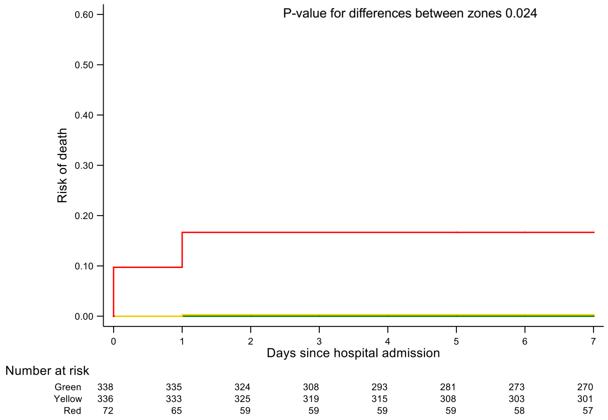

c. Subgroup of derivation cohort without malaria

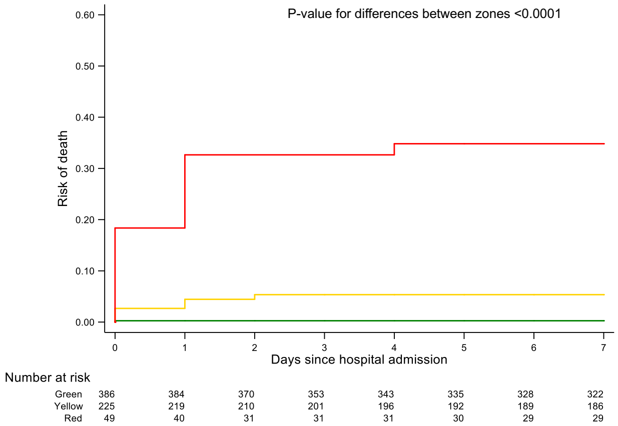

Derivation cohort included children enrolled between February 15, 2012 and October 31, 2012. Time-to-event curves were plotted based on Kaplan-Meier estimates. Wald test was used to calculate P-values for differences between zones.

Supplementary Fig. 6: Time-to-event analyses by sTREM-1 zones in the validation cohort for:

a. Whole validation cohort

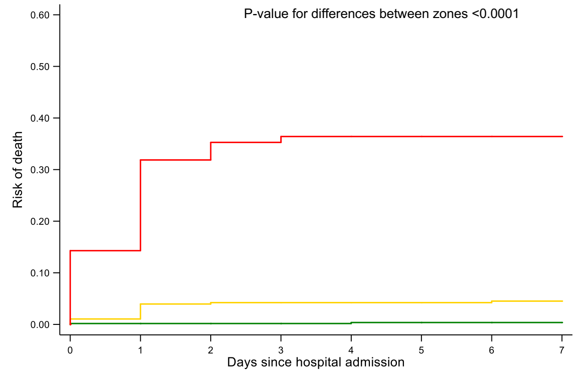

Number at risk

	0	1	2	3	4	5	6	7
Green	582	575	552	525	518	511	500	493
Yellow	381	375	357	344	337	333	329	328
Red	91	78	60	57	54	53	51	50

b. Subgroup of validation cohort with *P. falciparum* malaria

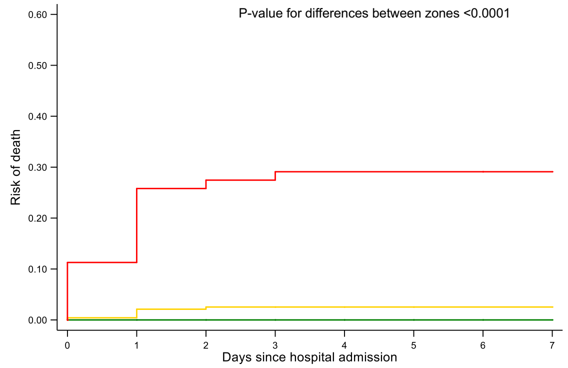

Number at risk

	0	1	2	3	4	5	6	7
Green	265	262	249	238	235	233	231	228
Yellow	238	236	228	220	214	212	210	210
Red	62	55	45	44	42	42	42	41

c. Subgroup of validation cohort without malaria

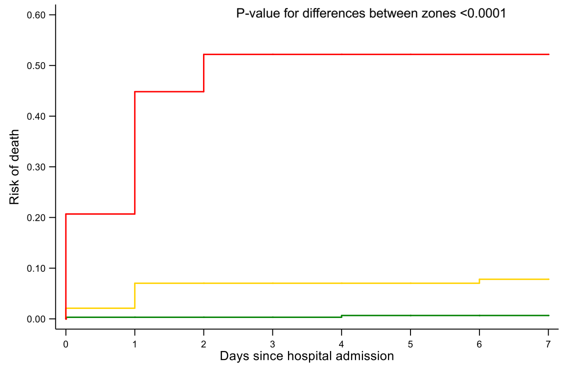

Number at risk

	0	1	2	3	4	5	6	7
Green	317	313	303	287	283	278	269	265
Yellow	143	139	129	124	123	121	119	118
Red	29	23	15	13	12	11	9	9

Validation cohort included children enrolled between November 1, 2012 and August 29, 2013. Time-to-event curves were plotted based on Kaplan-Meier estimates. Wald test was used to calculate P-values for differences between zones.

III. Supplementary references

1. Gelman, A. Scaling regression inputs by dividing by two standard deviations. *Stat Med* **27**, 2865-2873 (2008).
2. Pontrelli, G., *et al.* Diagnostic value of soluble triggering receptor expressed on myeloid cells in paediatric sepsis: a systematic review. *Ital J Pediatr* **42**, 44 (2016).
3. Bellos, I., *et al.* Soluble TREM-1 as a predictive factor of neonatal sepsis: a meta-analysis. *Inflamm Res* **67**, 571-578 (2018).
4. Saldir, M., *et al.* Endocan and Soluble Triggering Receptor Expressed on Myeloid Cells-1 as Novel Markers for Neonatal Sepsis. *Pediatr Neonatol* **56**, 415-421 (2015).
5. Stein, M., Schachter-Davidov, A., Babai, I., Tasher, D. & Somekh, E. The accuracy of C-reactive protein, procalcitonin, and s-TREM-1 in the prediction of serious bacterial infection in neonates. *Clin Pediatr (Phila)* **54**, 439-444 (2015).
6. Mazzucchelli, I., *et al.* Diagnostic performance of triggering receptor expressed on myeloid cells-1 and CD64 index as markers of sepsis in preterm newborns. *Pediatr Crit Care Med* **14**, 178-182 (2013).
7. Schlapbach, L.J., *et al.* Pancreatic stone protein as a novel marker for neonatal sepsis. *Intensive Care Med* **39**, 754-763 (2013).
8. Sarafidis, K., *et al.* Diagnostic utility of elevated serum soluble triggering receptor expressed on myeloid cells (sTREM)-1 in infected neonates. *Intensive Care Med* **36**, 864-868 (2010).
9. Chen, H.L., Hung, C.H., Tseng, H.I. & Yang, R.C. Soluble form of triggering receptor expressed on myeloid cells-1 (sTREM-1) as a diagnostic marker of serious bacterial infection in febrile infants less than three months of age. *Jpn J Infect Dis* **61**, 31-35 (2008).
10. Carrol, E.D., *et al.* The diagnostic and prognostic accuracy of five markers of serious bacterial infection in Malawian children with signs of severe infection. *PloS one* **4**, e6621 (2009).
11. Kevan, E.N., Simmons, J.R., Kocoshis, S.A., Cohen, M.B. & Rudolph, J.A. sTREM-1 and LBP in central venous catheter-associated bloodstream infections in pediatric intestinal failure. *J Pediatr Gastroenterol Nutr* **53**, 627-633 (2011).
12. Miedema, K.G., *et al.* The diagnostic value of CRP, IL-8, PCT, and sTREM-1 in the detection of bacterial infections in pediatric oncology patients with febrile neutropenia. *Support Care Cancer* **19**, 1593-1600 (2011).
13. Arzanian, M.T., *et al.* Association of serum soluble triggering receptor expressed on myeloid cells levels in malignant febrile neutropenic patients with bacteremia and fungemia. *Iran J Pediatr* **21**, 301-306 (2011).
14. Arizaga-Ballesteros, V., *et al.* Can sTREM-1 predict septic shock & death in late-onset neonatal sepsis? A pilot study. *Int J Infect Dis* **30**, 27-32 (2015).
15. Adly, A.A., Ismail, E.A., Andrawes, N.G. & El-Saadany, M.A. Circulating soluble triggering receptor expressed on myeloid cells-1 (sTREM-1) as diagnostic and prognostic marker in neonatal sepsis. *Cytokine* **65**, 184-191 (2014).

16. Erdman, L.K., *et al.* Combinations of host biomarkers predict mortality among Ugandan children with severe malaria: a retrospective case-control study. *PloS one* **6**, e17440 (2011).
17. Steyerberg, E.W. *Clinical Prediction Models: A Practical Approach to Development, Validation, and Updating*, (Springer, New York, NY, 2009).
