# Supplementary material for: Risk-stratification of febrile African children at risk of sepsis using sTREM-1 as basis for a rapid triage test
Source: Nat Commun. 2021 Nov 25;12:6832. doi: 10.1038/s41467-021-27215-6 (PMC8617180; doi:10.1038/s41467-021-27215-6)
Supplement: Supplementary file 3 — Reporting Summary [file 41467_2021_27215_MOESM3_ESM.pdf]

nature portfolio

Corresponding author(s): Kevin C. Kain

Last updated by author(s): Sep 30, 2021

Reporting Summary

Nature Portfolio wishes to improve the reproducibility of the work that we publish. This form provides structure for consistency and transparency in reporting. For further information on Nature Portfolio policies, see our Editorial Policies and the Editorial Policy Checklist.

Statistics

For all statistical analyses, confirm that the following items are present in the figure legend, table legend, main text, or Methods section.

n/a	Confirmed	
[ ]	[x]	The exact sample size (n) for each experimental group/condition, given as a discrete number and unit of measurement
[x]	[ ]	A statement on whether measurements were taken from distinct samples or whether the same sample was measured repeatedly
[ ]	[x]	The statistical test(s) used AND whether they are one- or two-sided<br>*Only common tests should be described solely by name; describe more complex techniques in the Methods section.*
[ ]	[x]	A description of all covariates tested
[ ]	[x]	A description of any assumptions or corrections, such as tests of normality and adjustment for multiple comparisons
[ ]	[x]	A full description of the statistical parameters including central tendency (e.g. means) or other basic estimates (e.g. regression coefficient) AND variation (e.g. standard deviation) or associated estimates of uncertainty (e.g. confidence intervals)
[ ]	[x]	For null hypothesis testing, the test statistic (e.g. F, t, r) with confidence intervals, effect sizes, degrees of freedom and P value noted<br>*Give P values as exact values whenever suitable.*
[x]	[ ]	For Bayesian analysis, information on the choice of priors and Markov chain Monte Carlo settings
[ ]	[x]	For hierarchical and complex designs, identification of the appropriate level for tests and full reporting of outcomes
[x]	[ ]	Estimates of effect sizes (e.g. Cohen's d, Pearson's r), indicating how they were calculated

Our web collection on statistics for biologists contains articles on many of the points above.

Software and code

Policy information about availability of computer code

Data collection	No commercial or open source software was used for data collection. Data were collected on paper Clinical Report Forms (CRF) and manually transcribed into Microsoft Excel (version 11.0) for future analysis (using STATA 15.1, as described below).
Data analysis	Statistical analyses were performed using Stata 15.1 (StataCorp, College Station, TX). Full SATA code used in the statistical analyses and figure generation in this manuscript are available from the authors on reasonable request. Source data is publicly available on Dryad and can be accessed using: https://datadryad.org/stash/share/MKYU_jq-yBhfIBziUY5BYcdDwGdlKGyruaUBdoSDS40

For manuscripts utilizing custom algorithms or software that are central to the research but not yet described in published literature, software must be made available to editors and reviewers. We strongly encourage code deposition in a community repository (e.g. GitHub). See the Nature Portfolio guidelines for submitting code & software for further information.

Data

Policy information about availability of data

All manuscripts must include a data availability statement. This statement should provide the following information, where applicable:

- Accession codes, unique identifiers, or web links for publicly available datasets
- A description of any restrictions on data availability
- For clinical datasets or third party data, please ensure that the statement adheres to our policy

Source data is publicly available on Dryad and can be accessed using: https://datadryad.org/stash/share/MKYU_jq-yBhfIBziUY5BYcdDwGdlKGyruaUBdoSDS40.
The data that support the findings of this study are also available from the authors.

Field-specific reporting

Please select the one below that is the best fit for your research. If you are not sure, read the appropriate sections before making your selection.

- [x] Life sciences
- [ ] Behavioural & social sciences
- [ ] Ecological, evolutionary & environmental sciences

For a reference copy of the document with all sections, see nature.com/documents/nr-reporting-summary-flat.pdf

Life sciences study design

All studies must disclose on these points even when the disclosure is negative.

Sample size	The original sample size consideration assumed a nested case-control design, defining in-hospital deaths as cases and using 3 survivors per case as matching controls. The recruitment of 2,475 children would result in 99 in-hospital deaths and more than 80% power to detect a standardized difference between cases and controls of 0.45 standard deviations using a Bonferroni correction that allowed this difference to be tested simultaneously for 13 biomarkers at a two-sided alpha of 0.0038 (0.05/13). The difference of 0.45 standard deviation units would correspond to a 40% difference in mean values of Ang-2, between a mean of 11,000 pg/mL in survivors and 15,600 pg/mL in children who died in-hospital, with a common standard deviation of 9,600 pg/mL.1 The current analysis reflects the original prospective cohort design rather than a nested case-control design, using in-hospital mortality from any cause up to 7 days as pre-specified primary outcome.
Data exclusions	Forty-two children were excluded from all analyses of biomarkers as no plasma sample was available (1.7%). An additional 376 children were excluded from comparative performance analyses of the 11 biomarkers (15.0%) as they had absconded or were transferred before or after 7 days (as shown in the flow chart in Fig.1).
Replication	Children enrolled up to Oct 31, 2012 were prospectively considered as part of the derivation cohort and children included after this date as part of the validation cohort. In addition, sTREM-1 analysis was validated in the derivation cohort based on 500 bootstrap samples with replacement (internal validation, as described in the main manuscript methods).
Randomization	This was a prospective cohort study in children aged 2 months to 5 years presenting to the emergency department with a history of fever in the past 48-hours or an axillary temperature >37.5ºC, and admitted to the Jinja Regional Hospital in Uganda between February 15, 2012 and August 29, 2013 according to the treating physician’s judgement. As described above, children enrolled up to Oct 31, 2012 were prospectively considered as part of the derivation cohort and children included after this date as part of the validation cohort (Steyerberg, E.W. Clinical Prediction Models: A Practical Approach to Development, Validation, and Updating, (Springer, New York, NY, 2009)
Blinding	This was a prospective cohort study which enrolled children consecutively presenting to the emergency department to the Jinja Regional Hospital in Uganda. Blinding was not relevant to the design of the prospective.

Reporting for specific materials, systems and methods

We require information from authors about some types of materials, experimental systems and methods used in many studies. Here, indicate whether each material, system or method listed is relevant to your study. If you are not sure if a list item applies to your research, read the appropriate section before selecting a response.

Materials & experimental systems

n/a	Involved in the study	
☒	☐	Antibodies
☒	☐	Eukaryotic cell lines
☒	☐	Palaeontology and archaeology
☒	☐	Animals and other organisms
☐	☒	Human research participants
☐	☒	Clinical data
☒	☐	Dual use research of concern

Methods

n/a	Involved in the study	
☒	☐	ChIP-seq
☒	☐	Flow cytometry
☒	☐	MRI-based neuroimaging

Human research participants

Policy information about studies involving human research participants

Population characteristics	We consecutively enrolled 2502 febrile children, with 1433 children up to Oct 31, 2012 included in the derivation cohort, and 1069 children from Nov 1, 2012 onwards included in the validation cohort. During the period of interest of 7 days, 2,039 children were regularly discharged or survived up to 7 days (81.5%), 95 children had died (3.8%), 337 absconded (13.5%), and 31 were transferred (1.2%). Table 1 shows a comparison of baseline characteristics of the 95 children who died up to 7 days with the 2,407 children who survived until regular discharge from hospital, abscondment or transfer. The age of children who died was 18.2 months and those who survived was 19.7 months (p=0.27). Of children who died, 56.8% were male and of those who survived, 54.9% were male. No genotypic information was collected or available for the cohort participants. Children who died within 7 days had a greater severity of illness (higher LODS score, higher lactate levels) and were less likely to be P. falciparum malaria positive. Supplementary Table 1 presents this comparison separately for derivation and validation cohorts. All children were evaluated promptly (Table 1) and treated according to national guidelines (Supplementary Table 2).
Recruitment	This was a prospective cohort study in children aged 2 months to 5 years presenting to the emergency department with a history of fever in the past 48-hours or an axillary temperature >37.5ºC, and admitted to the Jinja Regional Hospital in Uganda between February 15, 2012 and August 29, 2013 according to the treating physician's judgement. Patient enrolment occurred between 08:00 and 20:00. Patients presenting after 20:00 had a sample collected on the next day. Of 2,502 enrolled children, 11 presented after working hours, 8 of whom had follow-up to hospital discharge and were included in the analysis. As participants in this study were enrolled consecutively according to specified inclusion criteria, self-selection bias was not a factor in patient enrollment.
Ethics oversight	The study was approved by the Uganda National Council for Science and Technology, Makerere University Research Ethics Committee (Kampala, Uganda, REC Protocol # REF 2011-255), the University Health Network (Toronto, Canada, REB number 12-0039-AE), and was registered on clinicaltrials.gov (identifier: NCT04726826). The parent or caregiver of every study participant provided written informed consent.

Note that full information on the approval of the study protocol must also be provided in the manuscript.

Clinical data

Policy information about clinical studies

All manuscripts should comply with the ICMJEguidelines for publication of clinical research and a completedCONSORT checklist must be included with all submissions.

Clinical trial registration	ClinicalTrials.gov Identifier: NCT04726826
Study protocol	The study protocol of this study is available from the authors on reasonable request
Data collection	This was a prospective cohort study in children presenting to the emergency department with a history of fever in the past 48-hours or an axillary temperature >37.5ºC, and admitted to the Jinja Regional Hospital in Uganda between February 15, 2012 and August 29, 2013 according to the treating physician's judgement. Children enrolled up to Oct 31, 2012 were prospectively considered as part of the derivation cohort and children included after this date as part of the validation cohort.
Outcomes	Children were prospectively followed during their hospital stay. The main outcome was 7-day in-hospital mortality. Analyses of the comparative performance of the 11 biomarkers were based on children who did not abscond, were not transferred and did not have a missing plasma sample (n=2084). Remaining biomarker analyses were based on all children with an available plasma sample (n=2460); children without a plasma sample (n=42) were excluded throughout. To account for missing vital status in children who were transferred or absconded before 7 days we used multiple imputation (see Supplementary Information).<br>A secondary outcome was not included in any of the analyses and we focused only on the primary outcome of 7-day in-hospital mortality for all statistical analyses.
